# Vasogenic Cerebral Edema following CT Myelogram with Nonionic Omnipaque 300

**DOI:** 10.1155/2018/2761872

**Published:** 2018-05-22

**Authors:** Sara Khodor, Scott Blumenthal

**Affiliations:** ^1^University of South Florida, Tampa, FL, USA; ^2^South Florida Neurology Associates, Boca Raton, FL, USA

## Abstract

Computed Tomography (CT) with myelogram is a relatively safe procedure. It requires the use of nonionic contrast agents which, unlike ionic contrast agents, have been associated with low complication rates. We report a case of a 69-year-old female who developed diffuse bilateral cerebral edema following a lumber myelogram with the use of intrathecal nonionic contrast agent Omnipaque (Iohexol) 300. We were able to find one other reported case of cerebral edema following the use of intrathecal nonionic contrast agent in the literature.

## 1. Introduction

Vasogenic edema is the most common type of cerebral edema and develops as a result of breakdown of blood brain barrier and consequent albumin and fluid shift from the intravascular space and into the extravascular space. In turn, mass effect from vasogenic edema can cause reduced cerebral perfusion leading to ischemia and cytotoxic edema. Cerebral edema is not a well-known complication of intrathecal nonionic contrast material in CT myelograms. Intrathecal injection of nonionic contrast agents is known to be associated with adverse reactions related to pressure loss in the subarachnoid space and leakage at puncture site. These adverse reactions include headache, nausea, vomiting, and dizziness. Other reported complications of intrathecal nonionic contrast injections include aseptic meningoencephalitis, status epilepticus, and seizures.

## 2. Case Description

A 69-year-old Caucasian woman presents with confusion, headache, generalized weakness, ataxia, and increased agitation on the second day following a CT myelogram during which she received L2-L3 interspace lumbar puncture with fluoroscopic guided injection of 15 cc Omnipaque (Iohexol) 300, a nonionic water-soluble contrast material, in the prone position using a 20-gauge spinal needle. There were no complications during the procedure and after an adequate observation period the patient was discharged in a stable condition.

Her past medical history includes chronic back pain with spinal stenosis, diabetes, coronary artery disease, hyperlipidemia, colitis, gout, atrial fibrillation, and sick sinus syndrome. Her surgical history includes pacemaker placement, hysterectomy, and tonsillectomy. She has no known allergies. Her medication includes aspirin, allopurinol, amlodipine, colchicine, furosemide, gabapentin, metformin, pravastatin, metoprolol, pregabalin, and spironolactone.

On physical examination, the patient's vitals were stable. She was noted to be restless, agitated, and disoriented to place and had hyperactive deep tendon reflexes. She had no focal neurological deficits. Except for a mild elevation in WBC of 13.0, her laboratory values including CBC, CMP, urine analysis, and toxicity screen were all within normal limits.

**Figure 1:**
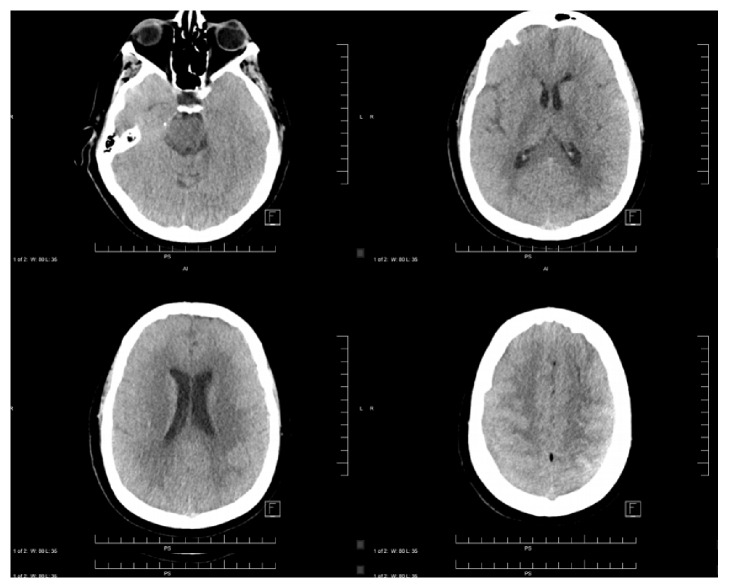
Supratentorial bilateral vasogenic edema with loss of all sulci and poor differentiation of white and gray matter.

**Figure 2:**
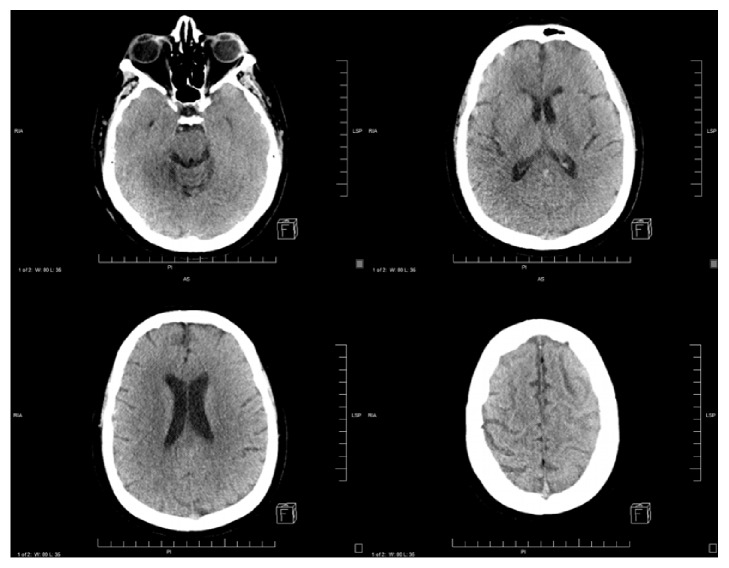
Improvement in vasogenic edema bilaterally with appreciation of sulci and improvement of white and gray matter differentiation.

A head CT revealed new supratentorial bilateral vasogenic edema with loss of all sulci and with poor differentiation of white and gray matter (Figure 1). There was no evidence of any compression of the quadrigeminal plate cistern or fourth ventricle on imaging. These findings were not visualized on previous imaging. Patient was admitted to the neurology intensive care unit (ICU) and was treated with mannitol, corticosteroids, and seizure prophylaxis. She was discharged in a stable condition after a 5-day admission with resolution of both clinical symptoms and radiographic findings of cerebral edema on head CT (Figure 2).

## 3. Discussion

### 3.1. Cerebral Edema

Cerebral edema is the accumulation of fluid in the brain as a response to cerebral injury such as trauma, infarction, hemorrhage, tumor, abscess, toxicity, and metabolism. Three types of cerebral edema include cytotoxic, vasogenic, and interstitial cerebral edema. Clinically there may be an overlap between the different types. Cytotoxic edema is the accumulation of fluid within glia, neurons, and endothelial cells most commonly due to hypoxia and usually starts within minutes of injury. Cytotoxic edema primarily affects the gray matter; however, ultimately white matter becomes involved as well [1]. Vasogenic edema, the most common type of cerebral edema, is secondary to the movement of albumin, other plasma proteins, and fluid from the intravascular space into the extravascular space. The breakdown of tight endothelial junction which compromises the blood brain barrier (BBB) facilitates this fluid shift [2]. This type of cerebral edema primarily affects the white matter [1]. Mass effect from vasogenic edema can cause reduced cerebral perfusion leading to ischemia and cytotoxic edema [2]. Interstitial cerebral edema is seen in hydrocephalus with obstruction of CSF outflow causing an increase in intraventricular pressure and movement of fluid to the paraventricular space [1].

Clinical signs of cerebral edema will depend on the location of the edema if focal edema is with focal neurologic symptoms. However, with generalized edema, there may be symptoms of elevated intracranial pressure including central herniation with alteration in mental status, abnormalities in extraocular movement and pupil size, breathing pattern changes, elevation in blood pressure, bradycardia, extensor plantar response, etc. Neuroimaging is useful in identifying the location and the type of cerebral edema. Serial CT or MRI studies are important in monitoring the resolution of edema after therapeutic intervention. On CT, edema appears hypodense or hypoattenuated; whereas on T2 MRI or Flair pulse series it appears hyperintense.

### 3.2. CT Myelogram and Contrast Agents

CT myelogram is performed with the use of intrathecal contrast agent for an improved and enhanced visualization of spinal cord lesions. The first-generation contrast agents were ionic and were associated with high rates of intravascular adverse reactions due to their high osmolality. As a result, they have been replaced, for the most part except for gastrointestinal and urologic procedures, with nonionic second-generation water-soluble agents that are associated with fewer adverse reactions. Although rare, contrast-induced neurotoxicity has been well documented with intravascular use of nonionic low osmolar contrast material [3–7]. Kocabay et al. discuss contrast-induced encephalopathy, seizures, cortical blindness, and focal neurological deficits in their retrospective analysis of 9 patients with contrast-induced neurotoxicity after administration of Iopromide, a nonionic low osmolar contrast agent for coronary angiography [3]. They recognized that neurotoxicity may depend on doses, route of administration, and procedure and that the mechanism behind the pathophysiology is controversial and had been attributed to disruption of BBB [3].

As for intrathecal use in CT myelography, first-generation high osmolality contrast agents could not be used due to neurotoxicity. In 1974, this procedure was transformed with metrizamide and years later with iohexol, both nonionic contrast agents [8]. A newer agent iodixanol which is a iso-osmolar, nonionic dimer is preferable for procedures during which the central nervous system, cardiovascular, or renal system is exposed to insult [9]. However, due to its increased cost, it is reserved for known high risk patients.

The adverse reactions associated with intrathecal injections of nonionic contrast agents are headache, nausea, vomiting, or dizziness, which may largely be attributed to pressure loss in the subarachnoid space resulting from intracranial hypotension from leakage at the puncture site. However, several case reports have described complications such as aseptic meningoencephalitis [10], status epilepticus [11], and seizures [12], after intrathecal nonionic contrast injections. In a retrospective analysis, Klein et al. report a risk of seizures in nonepileptic individuals and risk of status epilepticus in patients with epilepsy who have received Iopamidol myelography [13]. Kelley et al. described a case of cerebral edema in a 50-year-old female patient who presented with increased somnolence, headache, and visual changes a day following CT myelogram with Iopamidol [14]. Other reported cases have also described similar complications.

The pathophysiology for the development of neurotoxicity from intrathecal nonionic contrast injections is not well understood but has been linked to osmolarity disturbances [15], lipid solubility [16], or even direct toxicity [17] of these agents.

In our patient's case, she developed symptoms of cerebral edema manifested in altered mental status with reduced attention, agitation, and disorientation shortly after CT myelogram. Before the procedure, she was completely asymptomatic. This clinical presentation was supported with head CT findings of diffuse bilateral cerebral edema with absence of sulcal markings and poor differentiation of white and gray matter. We strongly suspect that the patient's presentation was a complication of the intrathecal use of nonionic Iohexol in CT myelogram.

### 3.3. Treatment and Management

General management of cerebral edema aims at optimizing cerebral perfusion, venous drainage, oxygenation, and minimizing cerebral metabolic demand [18]. Typical guidelines include maintenance of ICP < 20 mmHg and CPP 50 mmHg. There are various modalities which can be utilized to lower intracranial pressure and increase cerebral perfusion pressure. Elevating the head can decrease intracranial pressure (ICP) and usually recommended that the head is elevated at 30–60 degrees. Devices around the neck should be restricted as to not cause obstruction of venous outflow. Typically, patients are intubated for airway protection or to hyperventilate the patient to assist in lowering intracranial pressure. Mechanical ventilation should also be considered if the patient has hypoxia and hypercapnia as these conditions cause cerebral vasodilation and worsening edema. Controlled hyperventilation (PaCO2 25–30 mm Hg) can be used as a resuscitative measure for a short duration until more definitive therapies are instituted [18]. Other general measures including maintenance of intravascular volume with fluid management and vasopressors and adequate glycemic control are important for improved outcomes [18]. As for seizure prophylaxis, it is commonly used despite the lack of data proving its clinical benefit [18]. Specific measures such as corticosteroid therapy are used in selected patients; mostly those with vasogenic edema or brain neoplasm as it is thought to decrease capillary permeability of BBB [1]. Corticosteroids have not been shown to be effective in cytotoxic edema or stroke patients.

Medical management of cerebral edema may also entail the use of osmotic therapy, typically mannitol 20% or Hypertonic Saline. Mannitol acts as an immediate plasma expander by drawing fluid from extravascular to intravascular space, as a result improving cerebral blood flow which in turn causes cerebral vasoconstriction. Hypertonic saline acts as volume expander thus improving perfusion [19]. Loop diuretics are used sometimes in combination with osmotic agents to improve diuresis; however, their efficacy on cerebral edema is unknown and the risk of serious volume depletion may compromise cerebral perfusion. There is no strong data to support one osmotic agent over another.

In addition to the general management measures, our patient received mannitol. Close neuro exams were performed every 4 hours with gradual improvement. She was also treated with Keppra 500 oral BID for seizure prophylaxis in addition to dexamethasone 4 mg IV q6 h. Repeat CT scan showed resolution of edema (Figure 2). Patient was discharged after resolution of her symptoms and return to baseline in a stable condition after a 5-day admission to the neurology ICU.

## 4. Conclusion

Cerebral edema is not a well-known complication of intrathecal nonionic contrast material. We were only able to find one other similar case in the literature. Physicians should be aware of this rare complication after CT myelogram in order to implement early adequate therapeutic treatment to reduce patient's risk of mortality.
